# Activation of the DLPFC Reveals an Asymmetric Effect in Risky Decision Making: Evidence from a tDCS Study

**DOI:** 10.3389/fpsyg.2017.00038

**Published:** 2017-01-24

**Authors:** Daqiang Huang, Shu Chen, Siqi Wang, Jinchuan Shi, Hang Ye, Jun Luo, Haoli Zheng

**Affiliations:** ^1^School of Economics and Interdisciplinary Center for Social Sciences, Zhejiang UniversityHangzhou, China; ^2^Academy of Financial Research, Zhejiang UniversityHangzhou, China; ^3^School of Economics, Center for Economic Behavior and Decision-Making, Neuro and Behavior EconLab, Zhejiang University of Finance and EconomicsHangzhou, China

**Keywords:** risk preference, loss aversion, transcranial direct current stimulation, dorsolateral prefrontal cortex, asymmetric effect

## Abstract

The phenomenon of loss aversion (the tendency for losses to have a greater impact than comparable gains) has long been observed in daily life. Neurocognitive studies and brain imaging studies have shed light on the correlation between the phenomenon of loss aversion and the brain region of the prefrontal cortex. Recent brain stimulation studies using bilateral transcranial magnetic stimulation or transcranial direct current stimulation (tDCS) have obtained various results showing the causal relationship between brain regions and decision making. With the goal of studying whether unilateral stimulation can change participants’ risky decision making in the frames of gains and losses, we applied different polarities of tDCS over the regions of the right or left prefrontal cortex. We also designed a risk measurement table (Multiple Price List) to reflect the participants’ attitudes toward risky decision making via the crossover point including the frames of gains and losses. The results of our experiment indicated that the participants tended to be more risk averse in the gain frame after receiving left anodal tDCS and more risk seeking in the loss frame after receiving right cathodal tDCS, which was consistent with the hypothesis that the process of risky decision making was correlated with the interaction of multiple systems in the brain. Our conclusion revealed an asymmetric effect of right/left DLPFC when the participants faced gains and losses, which partially provided the neural evidence and a feasible paradigm to help better understand risky decision making and loss aversion. The current study can not only expand the traditional understanding of the behavioral preferences of humans in economics but also accommodate empirical observations of behavioral economists on the preferences of humans.

## Introduction

To determine how to address the complex problems of daily life, it is necessary to understand human decision making in the face of risk and uncertainty (Gurevich et al., 2009). For example, when offered a bet with an equal probability of winning or losing $100, most people would refuse to participate in the gamble. Behavioral economists and psychologists have demonstrated that a loss of $X is more aversive than a gain of $X; this phenomenon is called loss aversion and is closely related to risky decision making (Kahneman and Tversky, 1979, 1984; Tversky and Kahneman, 1992). Loss aversion also implies that when facing two options, for example, (A) a probability of 1.0 to win $3000 or (B) a probability of 0.8 to win $4000, most participants would choose the former. When faced with a different set of options, (A) a probability of 1.0 to lose $3000 or (B) a probability of 0.8 to lose $4000, most participants would choose the latter (Kahneman and Tversky, 1979).

In summary, compared with acquiring gains, people always have a much stronger intention to avoid potential losses, which is one of the most well-known and earliest reported anomalies discovered by economists (Tversky and Kahneman, 1981; Kahneman et al., 1990). In the middle of the 20th century, Markowitz (1952) and Williams (1966) studied this kind of abnormal behavior of investors in the stock market. Kahneman and Tversky (1979, 1984) studied the phenomenon of loss aversion and described the process in the framework of prospect theory: contrary to the traditional utility theory, the shape of the value function, which can be expressed as v(⋅), and the weighting function, which can be expressed as w(⋅), reflect the psychophysics of diminishing sensitivity in the theoretical framework of prospect theory. The value function is concave for gains but turned to be convex for losses, according to the theory, which indicates that the marginal impact of a change in outcome or probability diminishes with the distance from the relevant reference points (Kahneman and Tversky, 1979, 1984; Tversky and Kahneman, 1991, 1992). The characteristic of the value function, with a concave shape for gains and convex for losses, contributes to the risk aversion for gain but risk seeking for losses (Boorman and Sallet, 2009).

Evolutionary psychologists regard loss aversion as an instinct that evolved in humans (Breiter et al., 2001; Kenrick et al., 2009; De Martino et al., 2010; Sokol-Hessner et al., 2012). Most of the time, earnings and income would improve the living conditions of our ancestors, whereas unexpected losses could lead to death. For example, when crossing a dessert, additional drinking water would make people more comfortable, but lack of water would be fatal. In uncertain conditions, there are inevitably asymmetric expectations associated with gains and losses (McFarlane and Pliner, 1997). If this evolutionary claim is correct, then loss aversion is not a process of rational calculation but is more likely controlled by a neural structure in our brain that evolved long ago (Wilkinson and Klaes, 2012). Neuroeconomists and neuropsychologists have suggested that decision making under uncertainty is not a process of rational calculation based on the theory of classical economics but, rather, a heuristic process implemented in a brain region or neural structure to solve these problems in the face of uncertainty. Our brains and neural systems may form fixed neural circuits that are stimulated by survival-related events to ensure efficient decision making under similar conditions.

Recent neuroimaging studies have suggested that the risky decision making process of humans largely relies on the function of different brain regions. Breiter et al. (2001) applied functional magnetic resonance imaging (fMRI) and found that the amygdala and orbital gyrus were activated when the participants were faced with losses; Knutson et al. (2001) used a monetary incentive delay task to demonstrate the correlation between risky decision making and the striatum; Tom et al. (2007) revealed the neural basis of loss aversion, which attributes the decision process to the interaction and combination of different brain regions, including the ventromedial prefrontal cortex (VMPFC) and the ventral striatum (VS). Pammi et al. (2015) studied differences in neural loss aversion between depressed and healthy individuals to find that the two groups shared a brain network for value function approximation including the right ventral striatum, VMPFC, and right amygdala. As one of the most important brain regions in the cognitive process, the dorsolateral prefrontal cortex (DLPFC) has also been proven to be a region relevant to risky decision making. Mohr et al. (2010) used quantitative meta-analyses to reveal that the DLPFC was significantly activated in choice situations of risky decision making. Other evidence also revealed that the superior frontal gyrus (SFG), which is adjacent to the DLPFC, showed an increased activation during risky decision making (Engelmann and Tamir, 2009). All of the fMRI studies allowed researchers to identify the neural circuitry underlying different but related cognitive processes. However, non-invasive brain stimulation is also indispensable for allowing us to better understand the neurological process of decision making by showing us how the modulation of a specified brain region can directly impact our behavior.

Brain stimulation technologies, such as transcranial magnetic stimulation (TMS) and transcranial direct current stimulation (tDCS), have been widely used to study the neurological correlations of risky decision making and the changes in risk attitudes caused by stimulation in healthy participants (Gandiga et al., 2006). Compared with neuroimaging studies, brain stimulation studies are more focused on the cortex. Knoch et al. (2006) applied TMS to diminish the function of the right DLPFC of participants and found significant risk-seeking behavior in decision making in the TMS group. Fecteau et al. (2007a) showed that participants receiving right anodal/left cathodal tDCS adopted a risk-averse response style. Fecteau et al. (2007b) also indicated that simultaneous tDCS stimulation over the right and left DLPFC led participants to become much more risk averse than those with unilateral or sham stimulation. Boggio et al. (2010) found that after receiving left anodal/right cathodal tDCS, the older participants tended to choose the high-risk prospect more often than after receiving other kinds of stimulation. Weber et al. (2014) found that enhancing the activity of either the right or left DLPFC using tDCS did not change the risk attitudes of participants. Ye et al. (2015b) demonstrated that right anodal/left cathodal stimulation over the DLPFC caused risk-seeking behavior in situations of gains but risk-averse behavior in losses.

These varying results may be attributed to the use of different stimulation modes and experimental tasks (BART, “Balloon Analog Risk Task” or Roger’s Risk Task) (Rogers et al., 1999). In this study, we used tDCS to study the neural basis of loss aversion. By applying different polarities of tDCS over the right or left prefrontal cortex, we studied whether unilateral stimulation could change the participants’ responses to risky decision making in the face of gains or losses. We designed a simpler and clearer risk measurement table (Multiple Price List, MPL) based on a previous study, which reflected the participants’ degree of loss aversion in the risky decision making based on the crossover point (CP) in the frames of both gains and losses (Holt and Laury, 2002). According to the previous study, which used a risk task based on the design by (Holt and Laury, 2002; Ye et al., 2015a), we hypothesized that the DLPFC is responsible for risky decisions, and, furthermore, the right and left DLPFC areas play different roles in this process; the left DLPFC is sensitive to gains, whereas the right DLPFC is sensitive to losses.

The results of our experiment show that the tendencies of the participants are to be more risk averse in the gain frame after receiving left anodal tDCS and more risk-seeking in the loss frame after receiving right cathodal tDCS. The behavioral result is consistent with the loss aversion phenomenon in prospect theory. Our hypothesis that the process of risky decision making is correlated with the interaction of multiple systems in the brain can be partly confirmed (Rick, 2010). Our findings suggest that the left hemisphere is responsible for the evaluation in the gain frame, whereas the right hemisphere takes charge in the loss frame, which leads to risk aversion in choices involving sure gains and risk seeking in choices involving sure losses. This asymmetric effect improves our understanding of the different roles that the different regions of the DLPFC play in the process of decision making under risk, and it extends our knowledge of the left DLPFC’s function when facing gains and losses. In particular, our conclusion provides partial neural evidence and creates a useful paradigm to help us to better understand loss aversion in the framework of prospect theory.

## Materials and Methods

### Subjects

We recruited a total of 150 healthy students (82 females; mean age 21.3 years, ranging from 18 to 28 years) of Zhejiang University as the participants in our experiment. All participants met the following conditions: they were right-handed, naive to both tDCS and risk tasks, had no history of clinical impairments, psychiatric illness or neurological disorders. The participants were randomly assigned to the sham treatment (*n* = 30, 15 females) or to one of the four active treatments: left anodal tDCS (*n* = 30, 16 females), left cathodal tDCS (*n* = 30, 17 females), right anodal tDCS (*n* = 30, 20 females) and right cathodal tDCS (*n* = 30, 14 females). The participants received a fixed show-up fee of 30 RMB yuan (∼4.40 US dollars) plus the gain and loss from the task. On average, they received 32.34 RMB yuan (∼4.76 US dollars) from the task, ranging from 9 to 51 RMB yuan according to their performance and the computer program. The experiment was approved by the Zhejiang University ethics committee. The participants were asked to give written informed consent before starting the experiment. Some participants reported a slight itch during the stimulation, but none reported any adverse side effects involving pain on the scalp or headaches.

### Transcranial Direct Current Stimulation

Transcranial direct current stimulation applied weak but constant direct current over the scalp via two saline-soaked sponge electrodes (35 cm^2^). The constant current was delivered by a battery-driven stimulator (Multichannel, non-invasive wireless tDCS neurostimulator, Starlab, Barcelona, Spain), which was controlled by a Bluetooth system. The purpose of the tDCS was to induce cortical excitability of the target area without causing any physiological damage to the human body. Different orientations of the current have different effects on cortical excitability. In general, the anodal stimulation enhances cortical excitability and cathodal stimulation decreases the cortical excitability (Nitsche and Paulus, 2000).

**FIGURE 1:**
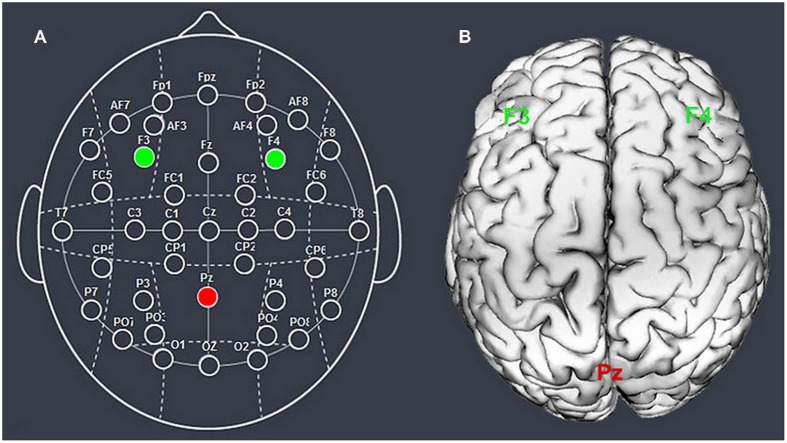
**Schematic and locations of the electrode positions.** Schematic of the electrode positions based on the EEG 10–20 system **(A)** and locations of the dorsolateral prefrontal cortex and the parietal cortex of the human brain **(B)**.

We chose the right F4/left F3 and Pz to place the electrodes, according to the international EEG 10–20 system (**Figure 1**). We stimulated the unilateral DLPFC instead of the DLPFC bilaterally because we aimed to distinguish the impact of the right or left DLPFC from the effects of changing the balance of activity across both DLPFCs. The parietal cortex was chosen to construct the current circuit together with the DLPFC because of its reasonable spatial and functional distance from our target region, which decreased the possibility of stimulation interaction or task interference (Simon et al., 2002).

**FIGURE 2:**
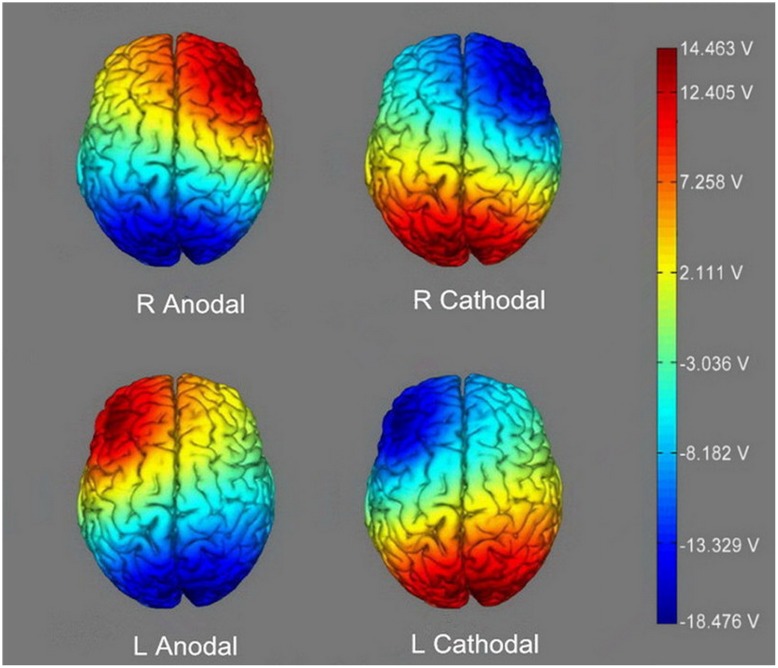
**The stimulation modes of the four active treatments.** The axis of this electronic potential diagram represents the input voltage of tDCS device, ranging from -18.476 V to 14.463 V. The deeper color represents the higher voltage and stronger current.

Participants assigning to different treatments received different stimulations. For right anodal stimulation, the anodal electrode was placed over F4, while the cathodal electrode was placed over Pz. For left anodal stimulation, the anodal electrode was placed over F3, and the cathodal electrode was also placed over Pz. For right cathodal and left cathodal stimulation, the placements were reversed. The anodal electrode was placed over Pz, and the cathodal electrode was placed over F4 or F3 (**Figure 2**). For sham stimulation, the same procedures were applied, but the current lasted only for the first 30 s. This brief duration of stimulation could hardly modulate cortical excitability, but the participants may have felt the initial itching and believed they were receiving stimulation. This kind of sham stimulation has been proved to be reliable (Gandiga et al., 2006). The constant current was 2 mA in intensity, with 15 s of ramp up and down, which has been shown to be safe and effective by previous studies.

For stimulation, the laboratory assistant first put the tDCS electrodes filled with conductive gel on a participant’s head and told him/her to rest on a sofa to stay calm. After 15 min of stimulation, the participant was asked to sit in front of a computer to do the task. After he/she finished the risky decision making task, the tDCS device was removed from the participant’s head.

### Task and Procedure

We designed an MPL, which was consisted of 10 choices (**Table 1**), to measure the degree of the participants’ risk aversion much simpler and clearer. Each choice has two different options, and in each option there are two realizations (A_1_ and A_2_ or B_1_ and B_2_) with different probabilities over the 10 rows. Option A is safer (the “safe” option) compared with option B (the “risky” option) in the same choice. Participants had to choose between the two options over the 10 rows in the two frames of gain and loss. After finishing the task, the computer randomly chose a row in each frame and decided the participant’s gain and loss according to the probabilities and the options he/she chose. For example, if the computer randomly chose row 3 in the gain frame, then the participant could gain 12 yuan (23 yuan) with a probability of 30% or gain 10 yuan (2 yuan) with a probability of 70% given he/she chose option A (option B). Similarly, if the computer randomly chose row 10 in the loss frame, then the participant would lose 12 yuan (23 yuan) with a probability of 100% given he/she chose option A (option B). Both gain and loss were included in the final payoff, encouraging the participants to earn as much as possible. The experiment was between-subject design to avoid the participants’ tendency of anchoring on the previous decision makings.

**Table 1:** Multiple price list.

Row No.	Option A		Option B	
	A_1_ = 12 Probability	A_2_ = 10 Probability	B_1_ = 23 Probability	B_2_ = 2 Probability
1	0.1	0.9	0.1	0.9
2	0.2	0.8	0.2	0.8
3	0.3	0.7	0.3	0.7
4	0.4	0.6	0.4	0.6
5	0.5	0.5	0.5	0.5
6	0.6	0.4	0.6	0.4
7	0.7	0.3	0.7	0.3
8	0.8	0.2	0.8	0.2
9	0.9	0.1	0.9	0.1
10	1	0	1	0


**FIGURE 3:**
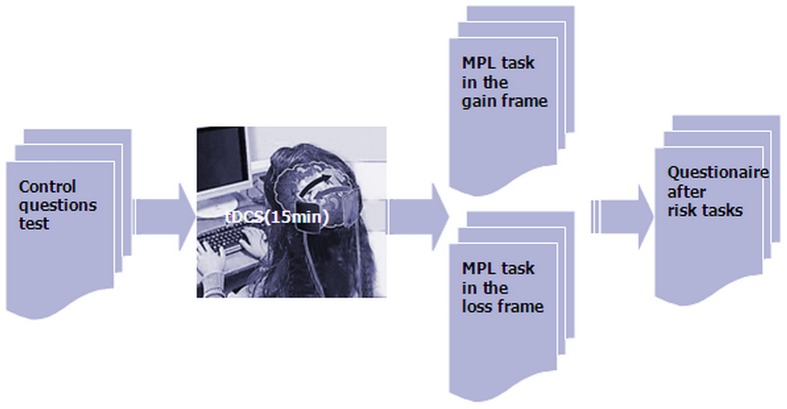
**Schematic representation of the experimental design**.

We used the experimental software z-Tree to present the two MPLs in the frames of gains and losses as well as to automatically calculate the final payoff (Fischbacher, 2007). Before the task, participants had to pass a control question test to ensure that they fully understood how their profit was decided. After the participants finished the task, they were asked to complete a questionnaire before they finally received their payment (**Figure 3**). The questionnaire contained questions about personal information, such as gender, age, income, consumption expenditure and self-assessment of risk preference.

### Data Analysis

In the first row of the gain frame, most participants, except extreme risk-takers, will choose option A because the expected value of option A is much higher than that of option B (10.2 yuan versus 4.1 yuan). In contrast, in the last row of the gain frame, all rational participants will choose option B because it provides a higher certain benefit. The expected value gap between option A and option B decreases from positive to negative as the row number increases. As a result, there will be one and only one CP for a rational participant with a consistent preference (Viscusi and Chesson, 1999; Holt and Laury, 2002). Option A is chosen before the CP and option B is chosen at or after the CP (extreme risk takers may choose option B in each row, thus the CP is set to 1). For example, if a participant chose option A in rows 1–6 and then chose option B in rows 7–10, then his/her CP was 7. The loss frame is in the same way. Most participants will choose option B in the first row and will choose option A in the last row in the loss frame. Then, option B is chosen before the CP, and option A is chosen at or after the CP. For the gain frame, the participant displayed a higher degree of risk aversion if he/she had a larger CP. In contrast, for the loss frame, the participant displayed a higher degree of risk aversion if he/she had a smaller CP. Therefore, the CP can be regarded as an index for depicting the participant’s degree of risk aversion.

We applied a mixed ANOVA using frame (gain vs. loss) as a within-subject factor and the treatment (right anodal, right cathodal, left anodal, left cathodal vs. sham) as a between-subject factor to detect the interaction effects in each condition. Then we conducted a one-way ANOVA to compare the participants’ CPs across the five treatments in the frame of gain as well as in the frame of loss. If the CPs of the four active treatments were significantly different from those of the sham treatment, we could speculate that the stimulation had changed the participants’ degree of risk attitude. Finally, we used a linear mixed effect model to estimate the possible impact of the treatment effects. The *post hoc* analyses were conducted using the Tukey HSD correction, and the critical level of significance was set at *p* < 0.05. All the statistical analyses were performed using SPSS software (version 20.0, SPSS Inc., Chicago, IL, USA).

## Results

**FIGURE 4:**
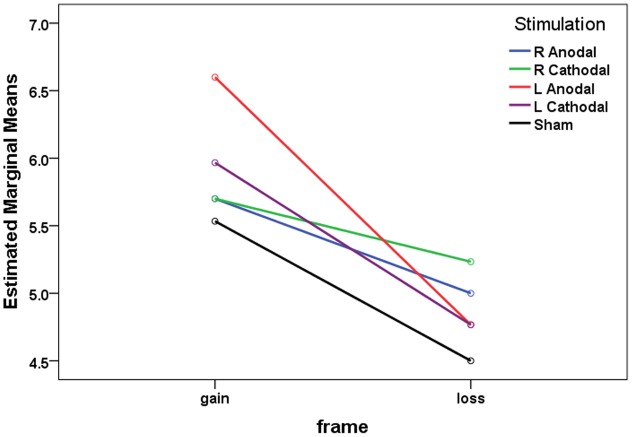
**The estimated marginal means of crossover points in both frames**.

We first applied a mixed ANOVA using frame (gain vs. loss) as a within-subject factor and the type of tDCS (right anodal, right cathodal, left anodal, left cathodal vs. sham) as a between-subject factor. The 2 (frame) × 5 (treatment) mixed-model ANOVA revealed that the main effect for frame was significant (*F*_1,145_ = 53.22, *p* < 0.001), indicating that the CP was significantly larger in the gain frame (mean = 5.900) than that in the loss frame (mean = 4.853, *p* < 0.001). The estimated marginal means are shown in **Figure 4**. A significant main effect for treatment was also observed (*F*_4,145_ = 2.907, *p* = 0.024). Crucially, significant interaction effects of frame by treatment were observed (*F*_4,145_ = 2.670, *p* = 0.035).

**FIGURE 5:**
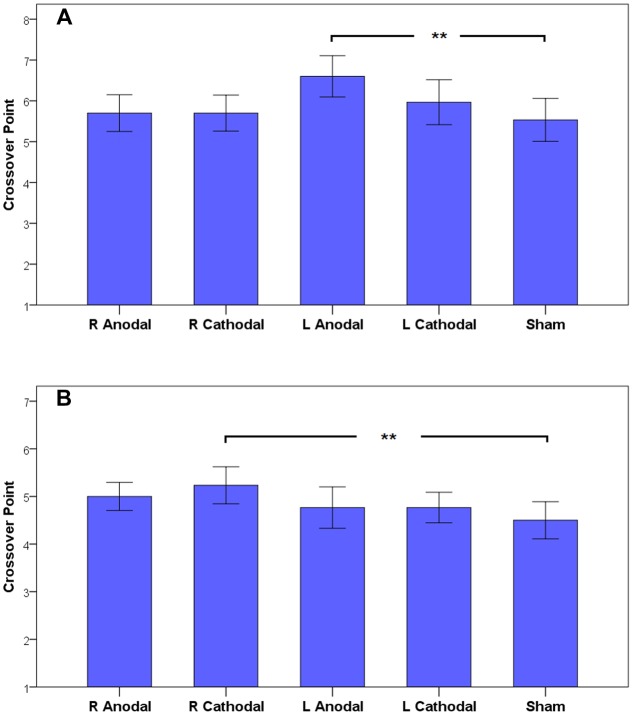
**The crossover points in the gain frame (A)** and the loss frame **(B)**. The error bars indicate 95% confidence intervals. The asterisks indicate statistical significance of differences between treatments.

Based on the results of the omnibus ANOVA above, we could focus on the specific analysis to reveal the effects of tDCS on risky choices. Simple effect analyses revealed a significant difference in the participants’ CPs in the gain frame and a marginal significant difference in the loss frame (one-way ANOVA; gain, *F*_4,145_ = 3.009, *p* = 0.020; loss, *F*_4,145_ = 2.348, *p* = 0.057). *Post hoc* analyses indicated that in the gain frame, the CPs of the participants receiving left anodal tDCS (mean = 6.60) were significantly larger than those of the participants receiving sham treatment (mean = 5.53, Tukey HSD, *p* = 0.019). In the loss frame, the CPs of the participants receiving right cathodal tDCS (mean = 5.23) were significantly larger than those of the participants receiving sham treatment (mean = 4.50, Tukey HSD, *p* = 0.037). No significant differences were found for the other active treatments compared to the sham group. This result indicates that the participants tended to be more risk-averse in the gain frame after receiving left anodal tDCS and tended to be more risk-seeking in the loss frame after receiving right cathodal tDCS. The CPs of the participants are shown in **Figure 5**.

The reaction time (RT) was also analyzed by applying a mixed-design ANOVA. Neither frame nor treatment had any significant effect on RT, which indicates that the RTs in each condition showed no significant difference. The mean RT and standard deviation are displayed in **Table 2**.

**Table 2:** The crossover point (CP) and reaction time (RT) of the participants.

		CP (Mean ±*SD*)	RT (Mean ±*SD*, second)
Gain	R+	5.70 ± 1.21	84.67 ± 22.63
	R-	5.70 ± 1.18	86.03 ± 18.54
	L+	6.60 ± 1.35	90.13 ± 21.72
	L-	5.97 ± 1.47	81.83 ± 16.24
	S	5.53 ± 1.41	81.97 ± 9.60
Loss	R+	5.00 ± 0.79	85.47 ± 29.96
	R-	5.23 ± 1.04	87.03 ± 24.87
	L+	4.77 ± 1.17	88.77 ± 21.37
	L-	4.77 ± 0.86	74.37 ± 26.26
	S	4.50 ± 1.04	80.06 ± 14.41


Finally, we applied a linear mixed-effect model with five dummy variables (four treatments and frame) and personal information as independent variables, with the CP as the dependent variable. The five dummy variables and the interaction of frame and treatment entered the model as the fixed effects, whereas the personal information like age and self-assessment risk as the random effects. The dummy variable R+ was equal to 1 if the participant received right anodal tDCS and was equal to 0 if the participant received the other treatments. Similarly, R-, L+ and L- were equal to 1 if the participant received right cathodal, left anodal and left cathodal tDCS, respectively. The dummy variable frame was equal to 1 if the participants facing gains, otherwise equal to 0. The coefficients and significance of the linear mixed-effect model are shown in **Table 3**. We can see that the frame has a significant impact to the participants’ degree of CP, which is consistent with the behavioral data. The significant coefficient of R- reveals that the right cathodal stimulation over right DLPFC may change the participants’ risky attitude. The interaction of frame and L+ also showed a marginal significance, which indicates that the influence of L+ stimulation was significant in the gain frame. The coefficients of personal information showed no significance.

**Table 3:** The coefficients and significance of linear mixed-effect models.

Parameter	Estimate	*SE*	*t*
Frame	1.033ˆ***	0.302	3.417
R+	0.488	0.305	1.468
R-	0.666ˆ**	0.306	2.174
L+	0.201	0.306	0.657
L-	0.259	0.302	0.855
Frame^∗^R+	-0.333	0.428	-0.779
Frame^∗^R-	-0.567	0.428	-1.325
Frame^∗^L+	0.800ˆ*	0.428	1.871
Frame^∗^L-	0.167	0.428	0.390
Age	-0.041	0.038	-1.093
Self-assessment of risk attitude	-0.072	0.082	-0.879


*^∗∗∗^p < 0.01, ^∗∗^p < 0.05, and ^∗^p < 0.1.*

## Discussion

Loss aversion exists in our daily life as an anomaly. For example, the return on equity is higher than the return on bonds, and consumers are far more sensitive to price increases than decreases. These phenomena or anomalies represent systemic deviations from classical economic theory, especially the basic hypothesis of the rational man in economics (Camerer et al., 1997; Camerer, 1998; Camerer and Loewenstein, 2004; Wilkinson and Klaes, 2012).

In addition to the mathematical proof, the psychological evidence for loss aversion has remained poorly understood. Some researchers have suggested that loss aversion appears to be moderated by affect (Trepel et al., 2005). Dhar and Wertenbroch (2000) reported that loss aversion was more pronounced for “hedonic goods” than “utilitarian goods”; the affective richness of consumption goods seemed to enhance differences in the perceived value of gaining versus losing these items.

Neuroimaging studies have indicated that loss aversion, as revealed by a risk-attitude measurement, has a neural basis involving the amygdala, orbital gyrus, and striatum or the interactional function between them (Knutson et al., 2001; Fukunaga et al., 2012; Sokol-Hessner et al., 2012; Krawczyk and D’Esposito, 2013). However, the prefrontal cortex is considered to be an important area for the emotional cognitive process of humans, which is inevitable to study its function during risky decision making. The prefrontal cortex is a large and heterogeneous brain region, and it appears that different regions may play different roles in decision making. Our tDCS study focused on the left/right DLPFC and applied a numerical task to observe the risk attitude of the participants. Our results showed that the left and right brain regions exhibit asymmetric functions in human loss aversion, especially in the gain and loss frames. To be specific, the participants became more risk averse in the gain frame when the excitability of their left DLPFC was enhanced by anodal tDCS stimulation, whereas the participants showed riskier patterns in the loss frame when the excitability of their right DLPFC was inhibited by cathodal tDCS. Concerning the hotly debated topic of whether loss aversion is a byproduct of a single system or results from the interaction of multiple systems in the brain, the results of our experiment add further evidence in support of the latter hypothesis, at least in the DLPFC. Our findings also support the reliability of neuroimaging results across different research groups (Cazzell et al., 2012; Bembich et al., 2014; Holper et al., 2014; Lin et al., 2014).

To further interpret the mechanism of risky decision making, we should pay attention to the emotional factors. Previous studies have shown that a risky decision process is generally associated with emotions. Specifically, the emotional processing occurred before the decision processing (Mohr et al., 2010; Martin and Delgado, 2011). tDCS has also been proven to be able to influence the emotional processing. For example, Peña-Gómez et al. (2011) demonstrated that tDCS over the DLPFC could change the perceived degree of the emotional valence for negative stimuli; Boggio et al. (2009) suggested that tDCS was effective in modulating the emotional process of pain. More recently, Engelmann et al. (2015) observed a disruption of neural valuation in risky decision processing caused by the anticipatory anxiety, revealing the neural mechanism of incidental anxiety on human risky decision making. Cohn et al. (2015) recruited financial professionals in different financial scenarios (boom or bust) to find that subjects were more fearful and more risk averse in the bust than in the boom condition, suggesting that fear may play an important role in countercyclical risk aversion. The close relationship between emotion and risky decision making has also been theoretically demonstrated by Phelps and her partners (Phelps et al., 2014). All of these studies strengthen the evidence linking risky decision making and the asymmetric mechanism that was found in our experiment.

Combined with previous neuroimaging and brain stimulation studies, the asymmetric effect observed in our experiment may further provide a new perspective on the phenomenon of loss aversion. As we know, the prefrontal cortex is often thought to be the primary site for cognitive regulation of emotion (Davidson et al., 2000), especially the right and left DLPFC, which play a variety of roles in different cognitive processes. Previous studies have provided numerous pieces of evidence that the right DLPFC is related to negative emotions such as depression, anxiety, fear and so on. Carlsson et al. (2004) demonstrated that the right DLPFC and the lateral orbitofrontal cortex were less activated by feared stimuli than to fear-relevant but non-feared stimuli. Shackman et al. (2009) showed that activation of the right DLPFC was related to behavioral inhibition. On the other hand, psychologists and economists have already suggested that negative emotions such as fear and anxiety could influence the sensitivity degree to losses (Camerer, 2005; Xu et al., 2009), which was consistent with our finding. In our experiment, the participants tended to be more risk-seeking after the cathodal stimulation over the right DLPFC, which may suggest that the right DLPFC is a loss-sensitive area that is more activated when making decisions about potential losses.

The left hemisphere is well known for its functions of logical analysis and rational calculation (McElroy and Seta, 2004). There have been few studies to demonstrate the relationship between the left DLPFC and decision making. However, some evidence has still shown that the left DLPFC region participates in the rewarding process associated with the dopamine (DA) system (Peciña et al., 2013). Combined with our previous results, we may speculate that the left DLPFC is a gain-sensitive area. When its cortical excitability was enhanced by anodal tDCS stimulation, the performance of our participants became more risk averse. In a word, the asymmetric mechanism of the right/left DLPFC can help us to better interpret the phenomenon of loss aversion.

The current method of unilateral DLPFC stimulation in our experiment provides a much more precise measurement of loss aversion, which may explain the differences between our results and other observations, and the method may help us to better understand the neural basis of loss aversion. Sellaro et al. (2015) showed that using an irrelevant brain region as a return electrode was possible; therefore, we placed the return electrode on Pz, for which there is no evidence that it is related to economic decision making. Unlike previous tDCS studies, we excluded the effect of tDCS attributed to the interaction results from modulating the excitability of bilateral brain regions and only showed the effect of a specific unilateral neural area. We distinguished between the effect of unilateral DLPFC stimulation and that of changing the activity across both DLPFCs. Our results reveal both a hemispheric asymmetry and a frame-dependent asymmetry in the performance of participants and suggest that the left hemisphere is responsible for the evaluation in the gain frame, whereas the right hemisphere takes charge in the loss frame. That is, when compared with the left DLPFC, the right DLPFC might be more sensitive to the change in monetary stimulation. This finding may help us to understand why the shape of the value function is concave for the quadrant of gains but convex for the quadrant for losses (Fox and Poldrack, 2008; Schonberg et al., 2011).

On the other hand, previous tDCS studies have measured risk attitudes using various tasks, and different results have been observed. We applied the MPL based on CPs originally designed by Holt and Laury (2002) to measure the risk attitudes of our participants. By using the original MPL design, we offered a simpler and clearer task table for the participants and reduced the possibility of switching back and forth from choice A to B more than once, which is inevitable in random-order designs. This strategy avoided potential inconsistencies in preferences, which is an important standard for rational decisions (Viscusi and Chesson, 1999). Because the MPL design has been popular in economic experiments to induce the utility function of risk attitudes, it is much more convenient to compare our experimental results with those of similar experiments.

A limitation of the current study was that we applied a between-subject design to avoid a potential learning effect in our experiment. Such a between-subject design may result in heterogeneity and diminished power of the data analysis. On the other hand, the decision making of the participants in the experiment relied on the expected value, and we still cannot evaluate the risk attitude precisely to model loss aversion. Future studies may focus on solving the trade-off between heterogeneity and the learning effect through more refined experimental designs, such as the BART or Roger’s task. Unlike the MPL task, these kinds of risky attitude measurements may have a more intuitive feeling for the participants. The frames of gains and losses in the current experiment were not counterbalanced, which might cause order effects. In addition, different brain regions, such as the striatum and the amygdala, can also be considered to help us explore the process of decision making better in the future.

## Conclusion

The current study explained why the majority of decision making tends toward risk aversion when facing choices that involve sure gains and risk seeking when facing choices that involve sure losses, and it also revealed both a hemispheric asymmetry and a frame-dependent asymmetry in the performance of participants confronting risk decisions. Our results provide further neural evidence for the loss aversion pattern and offer solid support for the reliability of neural imaging results in neuroscience. Both the MPL design using frames of gains and losses and the unilateral tDCS stimulation in our experiment provide much more precise measurements of risk attitude, demonstrating the particular functions of certain neural regions in human risky decision making. Although the study of decision making using cognitive neuroscience techniques is relatively young, a growing body of evidence suggests that decision making under risk is mediated by a network of cortical and limbic structures. Our experiment reveals that loss aversion has a controllable, reproducible and verifiable causal relationship with specific neural systems, providing further evidence for the understanding of prospect theory.

## Author Contributions

DH, SC, SW, JS, HY, JL, and HZ designed experiment; DH, SC, SW, JS, HY, JL, and H.Z performed experiment; SC analyzed data; DH drew figures; DH, SC, JL, and HZ wrote the manuscript; DH, SC, SW, JS, HY, JL, and HZ revised the manuscript and DH, SC, SW, JS, HY, JL, and HZ finally approved the version to be published.

## Conflict of Interest Statement

The authors declare that the research was conducted in the absence of any commercial or financial relationships that could be construed as a potential conflict of interest.
